# Congenital hereditary endothelial dystrophy with progressive sensorineural deafness: a case report of Harboyan syndrome

**DOI:** 10.5935/0004-2749.2023-0078

**Published:** 2024-09-16

**Authors:** Ezgi Karataş, Canan Aslı Utine

**Affiliations:** 1 Department of Ophthalmology, İbrahim Çeçen University Faculty of Medicine, Ağrı, Turkey; 2 Department of Ophthalmology, Dokuz Eylül University Faculty of Medicine, Izmir, Turkey; 3 Izmir Biomedicine and Genome Center, Izmir, Turkey; 4 Department of Ophthalmology, University of Naples Federico II, Naples, Italy

**Keywords:** Chromosomes, Corneal dystrophies, Exon, Genetic testing, Hearing loss, Hereditary/genetics, Mutation, Sensorineural, SLC4A11 gene

## Abstract

We present the case of a 37-year-old woman who underwent bilateral penetrating keratoplasty for congenital hereditary endothelial dystrophy at the age of 10 years. Over the subsequent 27 years, the patient’s vision slowly deteriorated. Our examination revealed decompensation of the right corneal graft. We addressed this with regraft surgery. We then learned that the patient had been suffering from progressive hearing loss since adolescence. Tonal audiometry revealed hearing per ceptive deafness of 25 dB, which was more prominent in the left ear. Because the patterns of progressive sensorineural hearing loss and congenital hereditary endothelial dystrophy have both been linked to the same gene, *slc4a11,* we tested our patient for mutations in this gene. The test was positive for a heterozygous *slc4a11* gene fifth exon mutation on chromosome 20p13-p12, which causes a frameshift. A combined clinical and genetic evaluation confirmed a diagnosis of Harboyan syndrome. After the genetic diagnosis of the disease, she was evaluated for the need for a hearing aid due to her hearing loss. The patient was also informed about genetic counseling.

## INTRODUCTION

Harboyan syndrome is a rare, benign, hereditary disorder that causes progressive sensorineural hearing loss (SNHL) and corneal dystrophy. Its estimated prevalence is less than 1 in 1,000,000. All confirmed cases have shown autosomal recessive inheritance^(1)^. Approximately 50% of the recorded instances manifest sporadically, whereas the remaining cases have been seen among the progeny of consanguineous unions. Fewer than 30 cases have been reported worldwide^(2)^.

The abnormalities associated with Harboyan syndrome are patterned by *slc4a11* expression in the corneal endothelium and inner ear, which encodes a transmembrane protein belonging to the SLC4 family of bicarbonate transporters^(3)^. The *slc4a11* gene is located on chromosome 20 (20p13-12) and is responsible for encoding a widely distributed electrogenic sodium-coupled borate transporter, commonly known as BTR1 or NABC1. This transporter plays a crucial role in maintaining cellular boron homeostasis. Malfunctions or deficiencies in BTR1 significantly impede cell development and proliferation.

Harboyan syndrome affects the corneal endothelial cells and is characterized by bilateral diffuse corneal edema, corneal opacification, blurred vision, visual loss, and nystagmus^(3)^. SNHL, as determined by tonal audiometry, is also characteristic of Harboyan syndrome. This form of hearing loss is post-lingual and slowly progressive. It generally begins between the ages of 4-19 and ranges from -30 to -60 dB.

We present the case of a 37-year-old female patient who underwent bilateral penetrating keratoplasty (PK) for congenital hereditary endothelial dystrophy (CHED) at the age of 10. Following graft decompensation 27 years after the initial operation, a reevaluation revealed Harboyan syndrome. We report the presentation, diagnosis, treatment, and outcomes of this case retrospectively. The case was treated in accordance with the Health Insurance Portability and Accountability Act and the tenets of the 2013 revision of the Declaration of Helsinki in Turkey^(4^,^5)^.

## CASE REPORT

**Figure 1:**
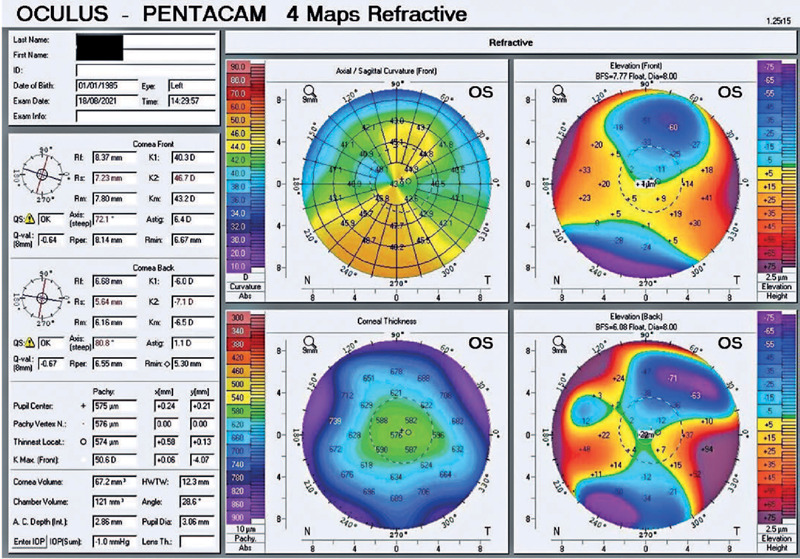
Tomographic examination results of a patient with Harboyan syndrome. The findings for the left eye indicated regular, within-the-rule astigmatism. Although the pachymetric measurement (576 µm) was somewhat higher than that of the right eye (Figure 3), there was no obvious clinical evidence of decompensation.

A 37-year-old woman presented at the Dokuz Eylül University Department of Ophthalmology Cornea Clinic with visual loss in her right eye, in which she had undergone corneal graft surgery 20 years earlier. Our examination revealed best corrected visual acuity of 3 logMAR and 0.3 logMAR in the right and left eyes, respectively. The left eye had a clear graft with no signs of decompensation, although the central corneal thickness was 574 µm (Figure 1). The intraocular pressure (IOP) was 15 mmHg in the right eye and 16 mmHg in the left.

The patient’s medical history showed that, at the age of 7, the patient had shown vision deterioration to counting fingers in both eyes. Both eyes showed nystagmus. The patient’s IOPs were within the normal range, excluding congenital or juvenile glaucoma. The ocular signs on slit-lamp assessment, including diffuse corneal edema and symmetrical thickening of Descemet’s membrane, led to a diagnosis of CHED. At the age of 10, the patient had undergone PK in both eyes at different sessions.

**Figure 2:**
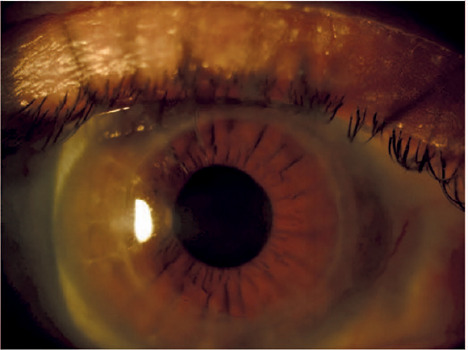
Photograph of the right eye of a patient with Harboyan syndrome at her last follow-up. The corneal regraft was clear and permitted detailed examination of intraocular structures. There was no evidence of decompensation or disease relapse.

**Figure 3:**
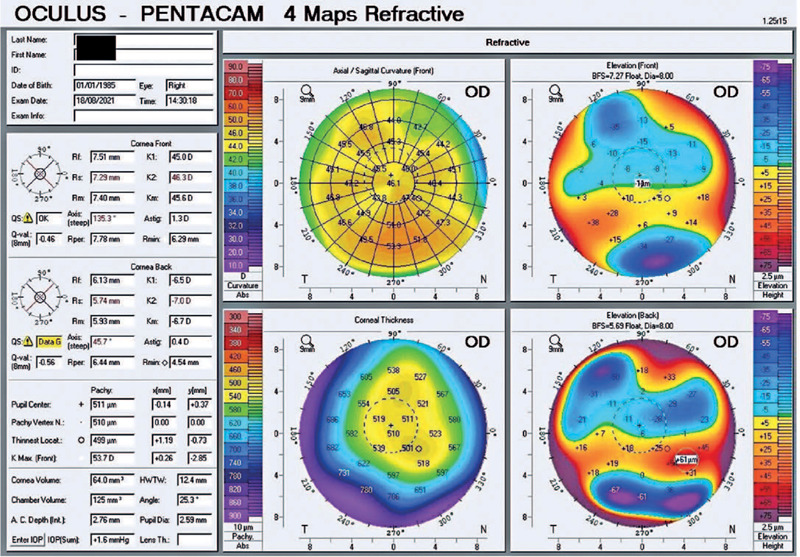
Tomographic examination of the right eye of a patient with Harboyan syndrome following a regraft procedure. The central pachymetric reading (510 µm) was normal but exhibited some irregular astigmatism of 1.3 D.

**Figure 4:**
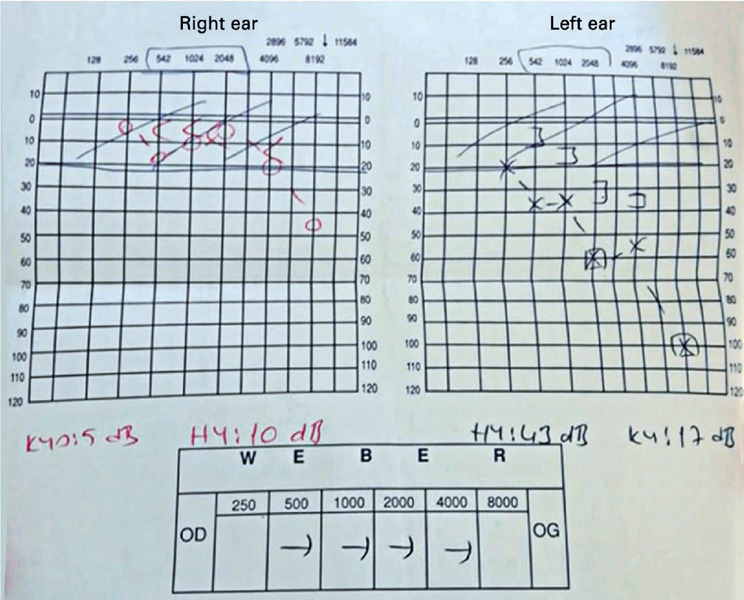
Tonal audiometry of a patient with Harboyan syndrome. This revealed hearing perceptive deafness of around 25 dB, which was more prominent in the left ear. A hearing aid for sensorineural hearing loss was recommended.

The patient was first admitted to our clinic with signs and symptoms of right eye corneal graft decompensation 27 years after her first PK surgery. Regraft surgery was performed on the right eye. This was well tolerated, and there was no subsequent recurrence of corneal edema (Figures 2 and 3). At the patient’s last visit, the visual acuity in both eyes was 0.48 logMAR. Further evaluation revealed that she had also been experien cing progressive hearing loss. This had begun during her teenage years. Tonal audiometry revealed that her left ear had greater hearing perceptive deafness than her right, at -25 dB (Figure 4).

Because progressive SNHL and CHED patterns have been associated with the same gene mutation, we evaluated our patient for the *slc4a11* gene mutation. Peripheral blood was used to obtain genomic DNA for genetic testing. A congenital hearing loss kit (Celemics, South Korea) and sequence analysis (Genomize, Turkey) software were used. Genetic pathology was classified according to the guidelines of the American College of Medical Genetics and Genomics. The test yielded positive results for the *slc4a11* fifth exon heterozygous mutation c.554_561del (chr20:3214819) (p. Arg185GlnfsTer4, rs869320721) on the 20p chromosome, which induces frameshifting.

## DISCUSSION

Harboyan syndrome was first described by Harboyan in 1971^(6)^. The ocular manifestations of the condition are identical to those in isolated CHED^(7)^. Corneal endothelial cells exhibit high levels of *slc4a11* expression^(8)^. This gene encodes a transmembrane protein in the SLC4 family of bicarbonate transporters; however, its role in transport remains unclear. Fibrocytes of the inner ear also have *slc4a11* gene-related membrane proteins. SNHL in patients with CHED can go unnoticed or remain asymptomatic before manifesting later in life^(9)^. Topical hyperosmolar solutions can be used to temporarily dehydrate the cornea, which may be advantageous in some patients. Corneal transplantation is the sole method for improving vision in patients with CHED. Research has found varying degrees of effectiveness in Descemet stripping endothelial keratoplasty for CHED but corneal transplantation via PK is usually the chosen therapy^(10)^. Hearing aids such as cochlear implants are recommended for SNHL.

The thick ascending limb of the renal loop of Henle expresses *slc4a11*. Multiple investigations have confirmed that *slc4a11* is important for thick ascending limb renal water reabsorption^(11^,^12)^. A decreased urine osmolarity and decreased concentrations of all electrolytes might result from this protein disruption. The major symptoms of Harboyan syndrome are corneal deterioration and hearing impairment. Renal abnormalities have also been reported but little is known about the incidence and features of these renal illnesses. A previous Harboyan syndrome case report found unilateral renal agenesis in the patient^(13)^. Another study found no polyuria or abnormal renal ion excretion among patients with Harboyan syndrome^(14)^. A potential correlation has been posited between the renal abnormalities reported in some patients with Harboyan syndrome and the presence of modifying genes or epigenetic alterations^(15)^. In the present case, no abnormalities in kidney function were found.

Harboyan syndrome has some value to the scientific community. The condition is a rare genetic illness that causes congenital corneal endothelial degeneration and progressive sensorineural deafness. As such, understanding the underlying genetic and molecular mechanisms of this syndrome may provide valuable insights into the development and function of the corneal endothelium and auditory system. Mutations in the *slc4a11* gene cause Harboyan syndrome and is therefore significant in the pathogenesis of the disorder. The transmembrane *slc4a11* protein transports borate and other ions across cell membranes. Learning about the function and control of this protein may help us understand ion transport pathways and their effects on corneal health and auditory function. Harboyan syndrome may also provide insights into corneal endothelium development and maintenance, molecular pathways, and gene functions.

### Summary of essential information

1. Genetic testing should be considered for all CHED patients.
2. The presence of isolated corneal edema throughout early infancy is a significant indicator of Harboyan syndrome.
3. The life expectancy of those with Harboyan syndrome is comparable to that of those without the condition.
4. The surgical prognosis of corneal transplantation in patients with Harboyan syndrome is generally favorable.
5. The prognosis of hearing loss in infants and young children with Harboyan syndrome is uncertain.
6. It is recommended that patients with Harboyan syndrome undergo regular follow-up testing of their renal function.
